# Chronic Stress and Headaches: The Role of the HPA Axis and Autonomic Nervous System

**DOI:** 10.3390/biomedicines13020463

**Published:** 2025-02-13

**Authors:** Aleksandar Sic, Marko Bogicevic, Nebojsa Brezic, Clara Nemr, Nebojsa Nick Knezevic

**Affiliations:** 1 Department of Anesthesiology, Advocate Illinois Masonic Medical Center, Chicago, IL 60657, USA; aca.smed01@gmail.com (A.S.); marko.bogicevic@midwestern.edu (M.B.); nebojsabrezic@gmail.com (N.B.); clara.nemr@midwestern.edu (C.N.); 2 Faculty of Medicine, University of Belgrade, 11000 Belgrade, Serbia; 3 Chicago College of Osteopathic Medicine, Midwestern University, Downers Grove, IL 60515, USA; 4 Department of Anesthesiology, University of Illinois, Chicago, IL 60612, USA; 5 Department of Surgery, University of Illinois, Chicago, IL 60612, USA

**Keywords:** chronic stress, migraines, tension-type headaches, cortisol, HPA axis, non-pharmacological treatments, autonomic nervous system, neuroendocrine regulation, stress-induced headaches

## Abstract

Chronic stress significantly influences the pathogenesis of headache disorders, affecting millions worldwide. This review explores the intricate relationship between stress and headaches, focusing on the dysregulation of the hypothalamic–pituitary–adrenal (HPA) axis and autonomic nervous system (ANS). Persistent stress could lead to neuroinflammation, increased pain sensitivity, and vascular changes that could contribute to headache development and progression. The bidirectional nature of this relationship creates a vicious cycle, with recurrent headaches becoming a source of additional stress. Dysregulation of the HPA axis and ANS imbalance could amplify susceptibility to headaches, intensifying their frequency and severity. While pharmacological interventions remain common, non-pharmacological approaches targeting stress reduction, such as cognitive-behavioral therapy, biofeedback, and relaxation techniques, offer promising avenues for comprehensive headache management. By addressing the underlying stress-related mechanisms, these approaches provide a sustainable strategy to reduce headache frequency and improve patients’ quality of life.

## 1. Introduction

Headaches, particularly migraines and tension-type headaches (TTH), are a pervasive health concern affecting millions of people worldwide [1]. These debilitating conditions not only cause significant pain and discomfort but also substantially impact daily functioning and quality of life [1]. Headaches are a clinical condition with varying frequencies and levels of associated disability and pain [2]. Recent studies indicate that over 50% of the world’s population experiences at least one headache per year. Among them, 14% suffer from migraines, 26% experience TTH, 4.6% have chronic headaches that occur on 15 or more days per month, and approximately 0.1% to 0.2% suffer from cluster headaches [3]. The widespread nature of these conditions underscores their considerable influence on overall well-being, quality of life, and economic burden on both personal and societal levels. Specifically, migraines alone affect over 580 million people worldwide, while TTH impacts nearly 965 million individuals. Both conditions are the leading causes of disability, significantly contributing to disability-adjusted life years (DALYs) and highlighting their substantial impact on public health [4].

While various factors contribute to the development and persistence of headaches, chronic stress has emerged as a critical element in their pathophysiology [1]. Stress can trigger headaches, exacerbate existing conditions, and even contribute to the transformation of episodic headaches into chronic forms [5]. Nearly 70% of individuals experience stress as a trigger for migraine attacks [5]. The experience of recurrent headaches itself becomes a source of stress, creating a vicious cycle that perpetuates the condition [6].

Recent research has shed light on the intricate mechanisms through which chronic stress influences headache disorders [7]. The hypothalamic–pituitary–adrenal (HPA) axis and the autonomic nervous system (ANS) play pivotal roles in this process [7]. Dysregulation of these systems due to persistent stress can lead to neuroinflammation, increased pain sensitivity, and vascular changes that contribute to headache pathogenesis [7].

While pharmacological interventions remain a cornerstone of headache management, there is a growing recognition of the importance of addressing underlying stress-related factors. Non-pharmacological approaches, including behavioral therapies, relaxation techniques, and lifestyle modifications, offer promising avenues for comprehensive headache care [1,6]. However, existing research on these therapies faces challenges, such as limited methodological standardization, inconsistent results, and insufficient focus on the stress-specific mechanisms underlying headaches. Moreover, their differential effectiveness across headache subtypes remains underexplored. Addressing these gaps is essential for enhancing the efficacy and practical implementation of non-pharmacological strategies.

This review addresses the gap in the literature by examining how the dysregulation of the HPA axis and ANS, driven by chronic stress, contributes to headache disorders. While previous research has investigated these systems individually, limited attention has been given to their combined role in the pathophysiology of stress-induced headaches. By exploring these interconnected mechanisms, we aim to provide insights into novel therapeutic strategies targeting stress-related dysfunction, offering a more sustainable and integrative approach to headache management.

## 2. Materials and Methods

A comprehensive literature search was conducted using PubMed, Scopus, Web of Science, and Google Scholar to gather relevant articles for this manuscript. The search focused on the relationship between chronic stress, the HPA axis, the ANS, and primary headaches primarily caused by stress. The keywords “chronic stress”, “HPA axis dysregulation”, “autonomic nervous system”, “primary headaches”, “migraine pathophysiology”, “non-pharmacological headache treatments”, and “cortisol and pain” were utilized. The search was restricted to peer-reviewed articles published between 2010 and 2024. However, older articles were also considered and included when deemed relevant to the topic. The inclusion criteria encompassed original research studies, systematic and narrative reviews, meta-analyses, and clinical guidelines, while non-peer-reviewed articles and publications in languages other than English were mainly excluded. Articles were initially screened based on their titles and abstracts, and those meeting the inclusion criteria were further assessed through a full-text review to ensure relevance to the manuscript’s objectives. All illustrations were created using online BioRender software (BioRender App, available at https://biorender.com).

The majority of the studies cited in this review are cross-sectional, limiting the ability to establish the exact causal relationship between chronic stress and headaches. While these studies provide valuable associations, longitudinal designs would offer insights into the temporal dynamics and progression of these conditions. In addition, the reviewed literature includes data from various global and regional contexts, yet disparities in cultural, healthcare, and socio-economic factors may influence the generalizability of the findings.

## 3. Chronic Stress and Dysregulation of the Stress Response Systems

### 3.1. Overview of the HPA Axis and ANS

The HPA axis, which involves the hypothalamus, pituitary gland, and adrenal glands, regulates glucocorticoid release in response to stress [8]. The ANS, consisting of sympathetic and parasympathetic divisions, governs functions such as the heart rate and blood pressure, which are essential for stress management and recovery [9]. Upon stress, the hypothalamus releases corticotropin-releasing hormone (CRH), which triggers adrenocorticotropic hormone (ACTH) secretion from the pituitary gland, prompting cortisol release from the adrenal glands to help cope with stress [10,11]. The sympathetic nervous system also activates the “fight-or-flight” response, releasing catecholamines like epinephrine, and the parasympathetic system helps return the body to a relaxed state once the stressor subsides by activating the vagus nerve to reduce the heart rate and blood pressure, lowering stress hormone levels through negative feedback mechanisms, dampening inflammatory processes, and promoting cellular repair to reestablish homeostasis and equilibrium [12].

When acute stress activates the HPA axis, cortisol is released to help the body respond effectively to the stressor. Once the stressor is resolved, a negative feedback mechanism is triggered, where elevated cortisol levels signal the hypothalamus and pituitary to decrease CRH and ACTH production, ultimately suppressing further cortisol release and restoring hormonal balance [13]. However, chronic stress disrupts the regulatory balance of the HPA axis, leading to its dysregulation. This dysfunction results in abnormal cortisol production, persistent activation, and associated physiological alterations [13,14]. Evidence from animal models highlights these effects, demonstrating adrenal hypertrophy and impaired glucocorticoid secretion, which further confirm the detrimental impact of chronic stress on the HPA axis [14].

### 3.2. Dysregulation of the HPA Axis and ANS in Chronic Stress

Chronic stress causes significant dysregulation in both the HPA axis and the ANS, leading to prolonged physiological alterations that contribute to various health issues, including headaches [15]. Prolonged activation of the HPA axis leads to elevated cortisol levels, which contribute to hippocampal atrophy, neuroinflammation, and heightened pain sensitivity—factors linked to chronic pain syndromes [15]. Research indicates that individuals with chronic pain often exhibit altered cortisol dynamics, such as low baseline cortisol and exaggerated responses to stress [11]. This dysregulation results in central sensitization, where persistent pain exacerbates stress responses, sensitizing individuals to non-painful stimuli and increasing the frequency and intensity of headaches, with the trigeminal system being particularly significant due to its role in transmitting pain signals from cranial structures and its direct involvement in the pathophysiology of migraines and tension-type headaches [12]. Chronic sympathetic activation further contributes to muscle tension and vascular changes, predisposing individuals to headaches. Sustained sympathetic activity leads to increased muscle tone in the head, neck, and shoulders, which can trigger tension-type headaches and cause blood flow changes closely linked to migraine development. These mechanisms amplify the frequency and severity of headaches, particularly in individuals with chronic stress [16]. In the early stages, dysregulation of both systems leads to increased pain sensitivity, which is typical of centralized pain disorders [17].

Additionally, abnormal regulation of the HPA axis, such as hyper- or hypocortisolism, alters downstream signaling, leading to mast cell activation and nociceptive afferent sensitization [17]. This process, termed “hyperalgesic priming”, results in central sensitization [17]. Prolonged activation of the HPA axis also induces neuroinflammation, contributing to oxidative stress and exacerbating pain responses through trigeminal pathway sensitization [18,19]. Cortisol also promotes gluconeogenesis, raising blood glucose levels to provide energy. However, when cortisol remains elevated over extended periods, it can lead to insulin resistance, contributing to metabolic disorders such as type 2 diabetes [20,21]. Moreover, chronic stress results in the dysregulation of immune function, characterized by an overproduction of pro-inflammatory cytokines and reduced anti-inflammatory responses. This imbalance weakens the immune system, increasing susceptibility to infections and autoimmune diseases [20,21].

Vascular changes, such as vasoconstriction followed by rebound vasodilation, play a key role in migraine attacks [21]. Vasoconstriction reduces cerebral blood flow, while rebound vasodilation leads to excessive dilation, which exacerbates headache symptoms [22]. Substances like nitric oxide and calcitonin gene-related peptide (CGRP) mediate these processes, linking vascular changes to migraine pathophysiology [23]. Prolonged activation of the HPA axis and the ANS not only exacerbates pain sensitivity but also impacts various other physiological systems, including the immune, gastrointestinal, and cardiovascular systems. HPA axis dysregulation impacts the immune system by shifting the balance between pro-inflammatory and anti-inflammatory cytokines, leading to a state of systemic inflammation. This is driven by persistent cortisol exposure, which initially suppresses immune responses but, over time, induces glucocorticoid resistance in immune cells, resulting in excessive pro-inflammatory cytokine production [18]. This widespread dysfunction, alongside heightened adrenergic activity from chronic sympathetic activation, alters autonomic cardiovascular regulation and amplifies pain perception, often leading to persistent headaches and other comorbidities associated with chronic stress [24].

Furthermore, the relationship between altered cortisol dynamics and brain plasticity complicates the pathophysiology of chronic stress. Chronic stress and sustained dysregulation of the HPA axis lead to changes in neural connectivity, particularly in the regions involved in pain processing, such as the thalamus and brainstem. These changes contribute to the heightened pain sensitivity observed in chronic pain conditions. Additionally, dysregulation of the ANS not only amplifies pain but also impairs the body’s ability to recover from stressful stimuli, creating a vicious cycle of pain and stress perpetuation. This impaired recovery arises from increased sympathetic activation that prolongs the stress response, while diminished parasympathetic activity fails to counterbalance this effect, leading to sustained adrenergic activity, systemic inflammation, and a delayed resolution of the stress-induced physiological changes. This dysregulation, involving the chronic activation of both systems, lays the foundation for the emergence of cyclical headache problems, where stress and pain reinforce each other, creating a vicious cycle. The heightened pain sensitivity, driven by altered neural connectivity and systemic inflammation, lowers the threshold for pain perception, making individuals more susceptible to headache triggers. This perpetuates the frequency and severity of headaches, as each episode of pain further amplifies the stress responses and disrupts autonomic regulation, ultimately locking the body into a persistent state of heightened pain and stress reactivity [25,26].

**Figure 1:**
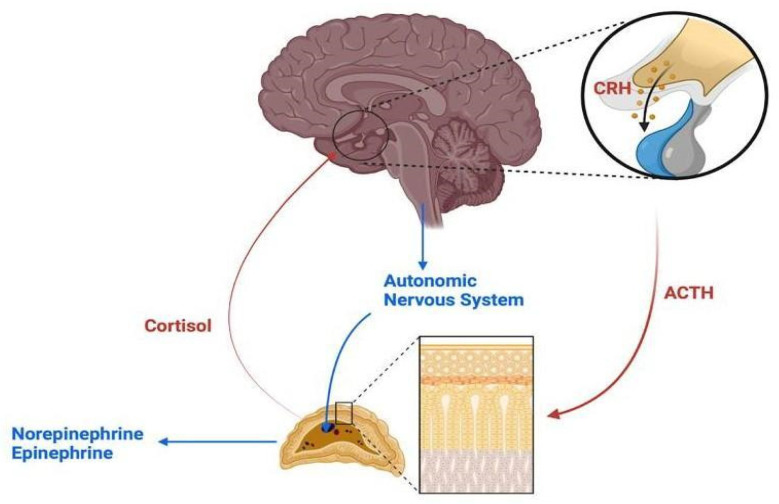
Interaction between the HPA axis and ANS stress response.

The complex interplay between the HPA axis and ANS stress responses, including feedback loops, is illustrated in Figure 1. Dysregulation of these systems in chronic stress plays a central role in influencing pain perception, as previously described.

### 3.3. Interactions Between the HPA Axis and ANS

The interplay between the HPA axis and ANS is central to mediating stress responses [27]. Sympathetic activation can influence HPA axis function via direct neural pathways, such as catecholamines enhancing CRH production and increasing ACTH secretion from the pituitary, thereby elevating cortisol levels [27]. This relationship explains how acute stress leads to prolonged hormonal changes that may contribute to headache disorders by promoting neuroinflammation, central sensitization, and vascular dysregulation, all of which lower the threshold for headache triggers and exacerbate pain sensitivity [16]. Chronic stress, characterized by hyperactivity in both systems, can result in maladaptive physiological responses, including lowered pain thresholds due to altered neurotransmitter systems involved in pain modulation [16]. The dysregulation of one system exacerbates dysfunction in the other, creating a feedback loop in which chronic headaches induce stress responses that further activate both systems [27].

The bidirectional communication between the HPA axis and ANS ensures that the body responds appropriately to stress. However, when both systems are chronically activated, maladaptive responses arise. Sympathetic activation increases the release of catecholamines, which, in turn, can reduce the parasympathetic regulation of inflammation, leading to sustained inflammatory processes that exacerbate pain and stress. Furthermore, the parasympathetic system’s inability to counterbalance sympathetic activation in chronic stress conditions can result in increased heart rate variability, which has been linked to impaired ability to regulate stress responses and heightened sensitivity to pain through mechanisms such as increased central sensitization, neuroinflammation, and reduced endogenous pain modulation [28].

It is beyond crucial to highlight the role of the vagus nerve, a key component of the parasympathetic nervous system, in regulating inflammation. In chronic stress, impaired vagal tone has been implicated in the development of conditions such as migraines and tension-type headaches. A deeper understanding of these mechanisms could pave the way for therapeutic interventions targeting the vagal system to reduce inflammation and modulate pain responses. Physiologically, the vagus nerve helps to counterbalance the heightened sympathetic nervous system activity that occurs during stress [28,29]. However, in states of chronic stress, vagal activity is often reduced, leading to a diminished capacity to regulate inflammation and control pain. This imbalance results in an overproduction of pro-inflammatory cytokines, which can promote the neuroinflammation and sensitization of pain pathways, contributing to the persistence of chronic pain conditions such as headaches [29,30].

## 4. Imbalance Between the Sympathetic and Parasympathetic Nervous Systems

### 4.1. Discrepancy Between the Sympathetic Nervous System (SNS) and the Parasympathetic Nervous System (PNS)

Chronic stress profoundly disrupts the balance between the SNS and PNS, resulting in maladaptive autonomic responses [31,32]. This imbalance contributes to the pathophysiology of headache disorders through mechanisms that go beyond general autonomic dysregulation. Increased SNS activity directly influences vascular dynamics, while heightened excitability in the trigeminal pain pathways lowers the headache threshold, intensifying headache frequency and severity [33]. Reduced PNS function exacerbates these effects by impairing pain inhibition and prolonging hyperalgesic states [34]. Furthermore, the diminished cholinergic anti-inflammatory pathway weakens neuroinflammatory regulation, perpetuating pain sensitization and contributing to the chronicity of migraines [35,36].

### 4.2. Cognitive, Emotional, and Neurochemical Dysregulation in Autonomic Imbalance

Chronic stress disrupts the balance between the SNS and PNS, contributing to cognitive and emotional dysregulation that exacerbates headache disorders. Excessive SNS activation, coupled with suppressed PNS activity, amplifies emotional states, such as anxiety, depression, and stress-related coping mechanisms, which, in turn, increase pain perception and the frequency and severity of headaches. This autonomic imbalance also worsens negative cognitive patterns, like rumination and catastrophizing, which further heighten stress responses and exacerbate autonomic dysregulation. The impact of SNS overactivation and PNS suppression extends to the brain regions involved in pain processing, such as the amygdala and prefrontal cortex. Increased SNS-driven amygdala activity and reduced PNS-mediated prefrontal regulation contribute to heightened pain sensitivity and emotional distress, creating a self-reinforcing cycle that worsens headache symptoms [26,37]. Additionally, chronic autonomic imbalance disrupts the balance of neurotransmitters such as serotonin, which plays a critical role in pain modulation. The sustained release of stress hormones like cortisol under excessive SNS activation promotes vasoconstriction, reducing the oxygen flow to the brain and potentially triggering headache episodes. The diminished anti-inflammatory and reparative effects of the PNS exacerbate these issues, prolonging pain and neuroinflammation. A persistent autonomic imbalance also induces a state of heightened vigilance, driven by increased SNS activity, which makes individuals more sensitive to pain and increases the likelihood of developing chronic headaches [1,38]. In summary, the interplay between SNS hyperactivity and PNS suppression not only disrupts autonomic regulation but also significantly impacts emotional, cognitive, and neurochemical processes, further contributing to the pathogenesis of chronic headaches.

### 4.3. Dominance of the SNS Under Chronic Stress and Reduced PNS Function

Prolonged exposure to chronic stress disrupts meningeal circulation and triggers trigeminal nociception through an ANS imbalance. This dysregulation contributes significantly to the development and progression of headaches [39]. Research shows that prolonged vasoconstriction and reduced endothelial nitric oxide (NO) production heighten the sensitivity of the meningeal vasculature. NO plays a critical role in vasodilation and neuronal excitability, but sympathetic overdrive reduces its production, leading to oxidative stress and vascular dysfunction [33]. This, in turn, sensitizes the trigeminal nociceptors to mechanical and chemical stimuli, contributing to the pathogenesis of migraines [33]. Furthermore, sustained SNS activation promotes the release of neuropeptides such as CGRP, which dilate the cranial vessels and further sensitize the trigeminal afferents, amplifying the migraine process [40]. Repeated trigeminal stimulation can lead to central sensitization within brainstem nuclei, including the trigeminal nucleus caudalis and higher-order pain-modulatory centers [41]. This results in increased neuronal responsiveness, expansion of the receptive fields, and heightened pain perception, facilitating the transition from episodic to chronic headache patterns [41]. Neuroimaging and electrophysiological studies support these mechanistic connections, revealing that individuals with chronic migraine and tension-type headaches frequently exhibit autonomic signatures that reflect ongoing sympathetic predominance. These diagnostic studies also show altered functional connectivity in the brain regions responsible for pain modulation, emotional regulation, and interoceptive awareness [42]. While these findings suggest a strong association between persistent SNS dominance, attenuated PNS function, and the reshaping of both peripheral and central neural mechanisms, they do not establish a direct cause-and-effect relationship. Instead, they highlight autonomic dysregulation as a likely contributor to the pathophysiology of chronic stress-related headache disorders.

Moreover, SNS dominance impacts the brain networks involved in pain perception and emotional responses. Chronic sympathetic hyperarousal alters functional connectivity within regions such as the amygdala, anterior cingulate cortex (ACC), insula, and medial prefrontal cortex (mPFC) [43]. Prolonged SNS activation has been associated with heightened amygdala activity, which amplifies pain-related signals and contributes to anticipatory anxiety regarding headache onset. This heightened amygdala response is linked to increased emotional distress, which may exacerbate headache symptoms. Conversely, dysfunction in the ACC and mPFC, regions critical for regulating emotional and cognitive responses, has been shown to coincide with insufficient PNS-mediated inhibition, potentially lowering the threshold for headache development and maintenance [44].

These persistent changes in brain networks contribute to a transition from episodic to chronic headaches, where autonomic dysregulation fuels both peripheral and central mechanisms [44]. Additionally, reduced vagal input in chronic headache patients is associated with dysfunction in the pain-modulating networks, contributing to a progression from episodic to chronic headaches [44,45].

These networks, which include brainstem nuclei, such as the locus coeruleus and the rostral ventromedial medulla, maintain a balance between the excitatory and inhibitory signals [46,47]. The imbalance in these signals leads to the amplification of pain signals at several neural checkpoints, contributing to a progression from episodic to chronic headaches [45,46]. Additionally, decreased vagal activity impairs the body’s ability to recover from acute stress events, which further promotes the chronicity of headaches. By limiting homeostatic balance after stress, ongoing PNS suppression creates a harmful physiological cycle that is characterized by increased oxidative stress, persistent low-grade inflammation, and heightened pain perception [48,49]. Therefore, chronic stress-induced vagal withdrawal is not only associated with headache disorders but also actively drives the underlying mechanisms of neuroinflammation, central sensitization, and impaired stress recovery, resulting in frequent, severe, and refractory headaches [50].

A recent review [51] highlights that interventions aimed at restoring autonomic balance may significantly impact headache-related pathophysiology driven by sustained sympathetic dominance and impaired parasympathetic function. Approaches such as non-invasive vagus nerve stimulation, mindfulness-based stress reduction, biofeedback, and cognitive-behavioral therapies have been explored to recalibrate the autonomic nervous system. By enhancing vagal tone, these therapies counteract sympathetic hyperactivity, reducing the pro-inflammatory and vasoactive states that sensitize the trigeminovascular pathways. This review further discusses the emerging evidence that promoting parasympathetic engagement can reduce CGRP release, alleviate meningeal vasoconstriction, and decrease neuronal hyperexcitability in the pain-processing regions of the brainstem and cortex. Beyond these acute effects, partial normalization of autonomic function has been linked to improved mood, reduced anxiety, and enhanced stress resilience, all of which disrupt the cycle of chronic pain. By gradually restoring autonomic homeostasis, these strategies reinforce inhibitory pain control mechanisms, stabilize inflammatory responses, and improve vascular regulation in areas associated with headache pain [51].

Overall, this growing body of evidence emphasizes the critical role of autonomic dysregulation in headache development and highlights the potential of targeting SNS–PNS imbalances for more effective, integrative headache management [52]. In summary, chronic stress leads to an imbalance between the SNS and PNS, creating a cycle of neuroinflammation, vascular dysregulation, and increased pain sensitivity—all of which can contribute to the persistence and exacerbation of headache symptoms [53,54]. Additionally, psychological factors, such as anxiety, depression, and stress-related coping mechanisms, further amplify this cycle. These emotional states not only affect autonomic balance but also influence pain perception and the severity of headache episodes [55].

## 5. HPA Axis Plasticity and Chronic Stress

The HPA axis is a fundamental neuroendocrine system that regulates physiological responses to stress [56]. Acute stress typically leads to the brief activation of the HPA axis, but chronic exposure to stressors can cause significant alterations in the HPA axis’ plasticity [31]. Plasticity is marked by structural and functional remodeling across the hypothalamus, pituitary gland, and adrenal cortex, as well as changes in glucocorticoid feedback sensitivity, particularly regarding cortisol [30]. In the context of chronic headaches, increasing evidence suggests that maladaptive HPA axis plasticity not only triggers stress-related signals but also lowers the threshold for pain generation and sensitization [39]. Dysregulation of the HPA axis in chronic headache patients has been linked to altered cortisol secretion patterns, which can contribute to heightened pain sensitivity and lower the pain threshold. Additionally, disruptions in the hypothalamus influence the release of stress-related hormones, including epinephrine and norepinephrine, which further exacerbate pain sensitivity and promote central sensitization [39].

**Figure 2:**
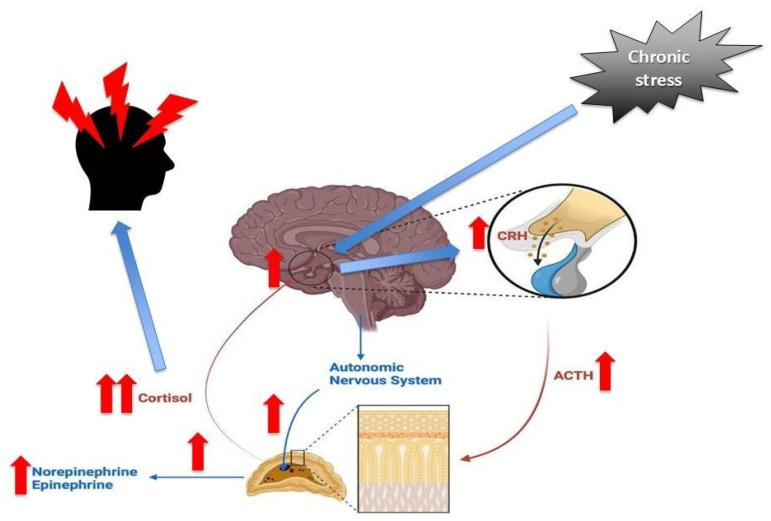
Dysregulation of the HPA axis in headaches influenced by chronic stress.

Below, Figure 2 represents the dysregulation of the HPA axis in headaches influenced by chronic stress, highlighting the cascade from stress to the hypothalamus, pituitary gland, adrenal glands, and the release of epinephrine, norepinephrine, and cortisol, ultimately contributing to headache development.

### 5.1. Structural and Functional Remodeling of the HPA Axis

Prolonged exposure to stress hormones induces neuroplastic changes in key brain regions that regulate HPA activity, such as the paraventricular nucleus of the hypothalamus (PVN), the hippocampus, and the prefrontal cortex (PFC) [10]. Chronic elevations in glucocorticoids can lead to dendritic remodeling and synaptic alterations in these areas, disrupting the balance between excitatory and inhibitory inputs that is critical for modulating the stress response [57]. As a result, the HPA axis becomes less responsive to negative feedback signals, leading to hypercortisolemia and an increased allostatic load [58]. This dysregulation is strongly associated with mood and anxiety disorders, and it has been implicated in persistent headache pathophysiology through heightened emotional reactivity and exacerbated pain perception [58].

### 5.2. Neuroendocrine Contributions to Headache Sensitization

Excess glucocorticoid exposure sensitizes the pain-processing networks within the trigeminal system and alters descending pain control pathways from the brainstem to the spinal trigeminal nucleus [39]. Studies show that chronic cortisol elevations modify synaptic plasticity in the pain-modulatory regions, diminishing the body’s endogenous analgesic capacity [41]. Dysregulated HPA axis function leads to the upregulation of pro-inflammatory mediators, such as interleukin 6 (IL-6) and neuropeptides like CGRP, which further lower the threshold for headaches. This is supported by the findings showing that patients with migraines have higher levels of IL-6, interleukin 8 (IL-8), and tumor necrosis factor (TNF-α) compared to healthy individuals, indicating that these pro-inflammatory cytokines may play a role in the development of a migraine [35,59]. This inflammatory cascade creates a feedback loop where elevated stress hormone levels drive inflammation and central sensitization, thereby fueling chronic headache syndromes [35,59]. The influence of chronic stress on neuroplasticity in pain-related brain areas such as the amygdala and hippocampus further contribute to the development of chronic pain [60,61]. Furthermore, genetic variations in the stress response system, such as polymorphisms in the *Methylenetetrahydrofolate Reductase Gene (MTHFR)* gene, may predispose individuals to enhanced susceptibility to stress-induced headache disorders, highlighting the role of individual genetic factors in this pathophysiology [60,61].

### 5.3. The Role of Oxidative Stress on Headaches

Chronic stress is known to induce oxidative stress and mitochondrial dysfunction, both of which play significant roles in the development of headaches [62,63]. Prolonged stress increases the production of reactive oxygen species (ROS) while impairing antioxidant defenses. This leads to damage to the neuronal lipids, proteins, and DNA [62]. This imbalance disrupts the trigeminovascular pathways and lowers the threshold for headache onset [63]. Mitochondrial dysfunction also enhances neuronal excitability and activates the NLRP3 inflammasome, which increases the production of pro-inflammatory cytokines such as IL-1β and TNF-α [64]. Furthermore, oxidative stress reduces NO bioavailability through the formation of peroxynitrite, impairing vasodilation and promoting vascular dysregulation. This mechanism plays a critical role in migraines and tension-type headaches [65]. The interaction between oxidative stress and mitochondrial dysfunction contributes to a pattern that leads to persistent headaches [66].

In conclusion, the interplay between oxidative stress and mitochondrial dysfunction creates a cascade of neuronal and vascular changes that lower the threshold for headache onset and perpetuate chronic headache conditions. Targeting these mechanisms through therapeutic interventions aimed at reducing oxidative stress and improving mitochondrial function may offer promising strategies for the prevention and management of headaches.

## 6. Genetic Factors in Stress and Headache Disorders

Genetic factors that influence stress response and HPA axis regulation have been the subject of several studies [67]. Some specific genes and their polymorphisms have been identified as potentially contributing to an individual’s susceptibility to chronic stress and headache disorders [67].

*Glucocorticoid Receptor Gene (NR3C1)*: The *NR3C1* gene encodes the glucocorticoid receptor (GR), which is crucial for the body’s response to cortisol. Certain polymorphisms in *NR3C1*, such as the *BclI* and *N363S* variants, have been associated with altered glucocorticoid sensitivity. Individuals with these variants may exhibit a diminished negative feedback response to cortisol, leading to sustained elevations in cortisol levels under chronic stress. This dysregulation of cortisol could increase susceptibility to stress-related conditions, including chronic headaches [68,69].

*Corticotropin-Releasing Hormone Receptor 1 Gene (CRHR1):* The *CRHR1* gene encodes the corticotropin-releasing hormone receptor, which is involved in initiating the stress response. Certain polymorphisms in *CRHR1,* such as the *rs110402* variant, have been associated with heightened sensitivity to stress and a greater risk of developing mood disorders, including those that often accompany chronic headache conditions like migraines [70,71].

*Catechol-O-Methyltransferase Gene (COMT):* The *COMT* gene plays a role in the breakdown of catecholamines, such as dopamine, norepinephrine, and epinephrine, which are involved in the stress response. Specific polymorphisms in the *COMT* gene, like *Val158Met*, have been linked to variations in stress reactivity and pain sensitivity. Individuals with the *Val/Val* genotype, which is associated with higher *COMT* activity, may experience heightened pain sensitivity and greater vulnerability to chronic pain conditions like headaches. In summary, while the *COMT Val158Met* polymorphism appears to influence pain sensitivity and may contribute to the pathogenesis of certain headache disorders, the relationship is complex and may be modulated by other genetic and environmental factors [72,73].

IL-6 and TNF-α: Chronic stress is often associated with increased levels of pro-inflammatory cytokines such as IL-6 and TNF-α. Polymorphisms in the IL-6 (*rs1800795*) and TNF-α (*rs1800629*) genes have been associated with altered cytokine production. Variants that lead to higher expression of these pro-inflammatory cytokines have been linked to chronic pain syndromes, including chronic headaches. These genetic variations may enhance the inflammatory response during stress, contributing to headache sensitization and chronicity [74,75].

*Methylenetetrahydrofolate Reductase Gene (MTHFR)*: The *MTHFR* gene is involved in folate metabolism and homocysteine regulation. Polymorphisms like *C677T* have been associated with higher levels of homocysteine, a known risk factor for vascular headaches, including migraines. Elevated homocysteine levels may contribute to endothelial dysfunction, blood–brain barrier disruption, and neurovascular inflammation, all of which can exacerbate headache pathology [76,77].

*Calcitonin gene-related peptide alpha (CALCA)* and *Transient receptor potential cation channel subfamily V member 1 (TRPV1)* genes are associated with the pathogenesis of headaches, particularly migraines. *CALCA* encodes a peptide that plays a key role in vasodilation and inflammation, two processes central to the development of headaches. Polymorphisms in this gene have been linked to a reduced response to botulinum toxin treatment in women with chronic migraines, suggesting its role in blood vessel dilation and pain perception. *TRPV1* is an ion channel that allows cation ions to enter cells, playing a key role in pain perception. Polymorphisms in the *TRPV1* gene are associated with increased pain sensitivity and response to botulinum toxin therapy, highlighting its significance in the inflammatory and nociceptive processes in migraines [78,79].

## 7. Stress and Headaches

Stress plays a central role in both the development and exacerbation of headaches, especially TTH, migraines, and their combined form (MigTTH) [80,81]. A study focusing on linking stress to migraines, TTH, and MigTTH frequency found that 31% of participants reported TTH, 14% experienced migraines, and 10.6% had MigTTH. Another study found that chronic migraine (CM) patients had significantly higher levels of perceived stress compared to the controls, with CM being a key contributor to perceived stress. Also, a significant negative correlation was found between perceived stress and migraine-specific quality of life (QOL), emphasizing the substantial impact of stress on CM patients’ well-being [82]. The most common types of stress-related headaches—TTH, migraines, and MigTTH—each have distinct characteristics, which require tailored approaches to treatment and management [80].

Cluster headaches, while less common than migraines and tension-type headaches, are characterized by excruciating, unilateral pain typically centered around the eye or temple, often described as a piercing or burning sensation [83]. Attacks are usually accompanied by autonomic symptoms such as lacrimation, nasal congestion, eyelid swelling, or facial sweating, and they occur in clusters over weeks or months, separated by periods of remission. Unlike other primary headaches, stress appears to have a limited role in the development or exacerbation of cluster headaches. Instead, these headaches are more closely associated with disruptions in circadian rhythms and hypothalamic dysfunction [83]. Table 1 represents the global prevalence of primary headaches.

### 7.1. Tension-Type Headaches

Tension-type headache (TTH) is the most prevalent neurological disorder globally, characterized by recurrent, mild to moderate bilateral pain often described as a pressing or tightening sensation resembling a band-like tightness around the head [84,85]. Unlike migraines, TTH is not typically associated with nausea or a heightened sensitivity to light and sound. Diagnosis relies on the clinical history and the exclusion of secondary causes, following the International Classification of Headache Disorders, third edition (ICHD-3) guidelines [84,86].

Chronic stress plays a significant role in the development and exacerbation of TTH. Psychosocial stress can lead to sustained muscle tension in the head, neck, and shoulders, increasing the likelihood of TTH episodes [87]. Additionally, chronic stress contributes to central sensitization, a key mechanism underlying TTH, where the central nervous system becomes more responsive to pain [88]. Prolonged exposure to stress can induce neuroplastic changes that amplify pain intensity and frequency, highlighting the bidirectional relationship between stress and TTH [88,89].

TTH is classified into episodic and chronic forms. Episodic TTH, typically lasting 30 min to 7 days, may be influenced by transient stressors, whereas chronic TTH, occurring on 15 or more days per month, is more strongly linked to persistent stress and associated conditions like anxiety and depression [90]. These psychiatric comorbidities not only exacerbate the symptoms but also perpetuate the cycle of stress and pain, complicating treatment and increasing the burden of disability [90,91].

Chronic stress may also indirectly contribute to medication overuse headache (MOH), a frequent complication in individuals with chronic TTH who excessively use analgesics or triptans. This phenomenon further intensifies headache frequency and severity, underscoring the importance of addressing stress management in the treatment of TTH [92,93].

In conclusion, chronic stress significantly impacts the pathophysiology and clinical course of TTH, amplifying central sensitization and perpetuating a cycle of pain and dysfunction. Addressing stress through targeted interventions may be critical for effective TTH management.

### 7.2. Migraines

Migraines are primary headaches that rank as the second leading cause of disability worldwide [94], with an estimated global prevalence of over 1 billion people [95]. Migraines are characterized by intense, throbbing pain, often on one side of the head, and typically last from 4 to 72 h. These headaches are commonly accompanied by symptoms such as nausea, vomiting, and a heightened sensitivity to light (photophobia) and sound (phonophobia) [96]. Some individuals also experience an aura, which can involve visual disturbances, such as flashing lights or blind spots, or sensory changes, such as tingling or numbness, although over 75% of migraines occur without aura [96]. Migraine attacks can be triggered by various factors, including hormonal changes, stress, lack of sleep, certain foods, and environmental stimuli [96]. Risk factors for developing migraines include a family history of the condition, female gender (due to hormonal influences), younger age (with onset typically between 18 and 44 years), and high levels of stress or anxiety. Other triggers may include caffeine consumption, dehydration, and sleep disturbances [97,98,99].

The pathophysiology of migraines is complex and not fully understood, but it is believed to involve both vascular and neuronal mechanisms [99]. A primary theory suggests that a process known as cortical spreading depression (CSD) triggers a cascade of events that lead to the release of neurotransmitters such as serotonin, CGRP, and substance P, which contribute to inflammation and vasodilation in the brain [99,100]. This neurovascular hypothesis highlights the interaction between blood vessels and nerve cells in the brain. Furthermore, imbalances in the brain’s pain-processing pathways, particularly in the trigeminal nerve system, are thought to play a key role in migraine pathogenesis [101]. This dysfunction causes the brain to become hypersensitive to stimuli, leading to intense pain and other sensory disturbances characteristic of a migraine episode [101].

Migraine episodes significantly impact daily life, reducing work productivity, limiting social activities, and affecting emotional well-being [101,102]. Chronic migraines, defined as headaches occurring 15 or more days per month for at least three months, are associated with frequent, severe attacks and a substantially decreased quality of life. Chronic migraine sufferers often have higher rates of comorbidities such as depression and anxiety, both of which are exacerbated by chronic stress [103]. Chronic stress contributes to this burden by intensifying migraine severity and frequency through mechanisms such as HPA axis dysregulation and increased central sensitization, which amplify neuronal hyperexcitability and lower the threshold for migraine triggers [104]. Additionally, chronic stress perpetuates a cycle of heightened sensitivity to migraine triggers, including sleep disturbances, emotional stress, and hormonal fluctuations, leading to recurrent attacks and greater disability. This stress-migraine cycle is further compounded by neuroinflammatory processes and vascular dysregulation, both of which are modulated by stress-induced oxidative and mitochondrial dysfunction [103,104]. Migraine–tension-type headache (MigTTH) represents a unique headache disorder characterized by overlapping features of migraines and TTH, such as the pulsating, unilateral pain of migraines and the bilateral, pressing sensation typical of TTH. While limited epidemiological data exist on MigTTH, some studies have highlighted its prevalence and the shared pathophysiological mechanisms between migraines and TTH, including the role of stress in central sensitization and autonomic dysregulation [105].

Central sensitization is thought to play a critical role in the convergence of migraine symptoms. Dysregulation in the pain-processing pathways involving the trigeminovascular system, along with heightened muscle tension in the head, neck, and shoulders, likely contributes to the development of this condition [106]. Moreover, chronic stress, sleep disturbances, and psychiatric comorbidities, mainly anxiety and depression, are recognized as key risk factors that exacerbate both the frequency and severity of MigTTH episodes [107].

Below, Table 2 summarizes the risk factors and main clinical features of primary headaches.

## 8. Treatment of Stress-Related Headaches

The treatment of stress-related headaches, including TTH, migraines, and MigTTH, involves both pharmacological and non-pharmacological approaches that are tailored to the specific characteristics of each type of headache [110].

### 8.1. Pharmacological Treatment

The pharmacological treatment of stress-related headaches focuses on both acute symptom relief and the prevention of future episodes [111].

When treating TTH, the acute treatment typically includes simple analgesics like acetaminophen, ibuprofen, or aspirin [112]. These are effective for relieving mild to moderate pain. In cases of more frequent or chronic TTH, combination medications containing analgesics and caffeine, such as Excedrin, may be prescribed to enhance pain relief [113]. For preventive treatment, TCAs, but mostly amitriptyline, are commonly used, as they help reduce headache frequency by modulating central pain pathways [112]. CGRP monoclonal antibodies have recently emerged as a viable option for TTH prevention [96]. Moreover, tizanidine, a muscle relaxant, can be helpful for patients who experience muscle tension in the head, neck, and shoulders, contributing to the headache. However, there are some limitations to be aware of, as triptan is not to be prescribed for patients with a history of cardiac diseases or hypertension [112].

When treating migraines, the focus is both on relieving the symptoms during an attack and preventing future episodes. For acute attacks, triptans are the first-line treatment [114], although they are considered less effective than triptans in providing symptom relief. They generally offer comparable or even superior outcomes when contrasted with NSAIDs, ASA, and acetaminophen [115]. Combining triptans with ASA or acetaminophen or using alternative administration methods like injectables may provide slightly enhanced results over the standard oral triptan tablets [115]. They bind to the serotonin receptors, constricting blood vessels and reducing the inflammatory response. NSAIDs like ibuprofen and naproxen are also used for pain relief, while antiemetics help manage the nausea and vomiting associated with migraines [115,116]. For migraine prevention, beta-blockers, anticonvulsants, and CGRP inhibitors are recommended, as they help reduce the frequency and intensity of the migraines by targeting the neurological pathways involved in migraine pathophysiology [114]. Valproate (500–1500 mg/day) and topiramate (200 mg/day) have been shown to effectively decrease the frequency of migraines [117]. However, newer drugs like CGRP monoclonal antibodies are gaining popularity due to their targeted action and ability to reduce migraine episodes by blocking the CGRP receptor [112]. In MigTTH, treatment typically combines both migraine and TTH therapies [118]. Managing migraines in patients with comorbid conditions presents significant challenges for clinicians. Conditions such as chronic pain syndromes, psychiatric disorders (e.g., depression, anxiety, and bipolar disorder), and medication overuse can complicate migraine treatment. These patients often require a multidisciplinary approach that incorporates both pharmacological and non-pharmacological strategies tailored to their specific needs [119].

Furthermore, pharmacological treatments for headaches, including triptans, TCA, ergotamines, NSAIDs, and opioids, can lead to various side effects, which include dizziness, cardiovascular issues, gastrointestinal problems, respiratory depression, and a risk of dependence [120]. Additionally, antidepressants may increase the risk of serotonin syndrome, highlighting the need for careful monitoring and appropriate medication selection [121,122]. However, it is important to note that these treatments primarily address the symptoms and do not causatively alter the underlying mechanisms contributing to the headache

### 8.2. Non-Pharmacological Treatment

Non-pharmacological treatments for headaches are often considered due to several factors, including avoiding medication overuse, managing the side effects of certain medications, addressing tolerance towards common pharmaceutical therapies, considering contraindications for pharmacological treatments, evaluating cost-effectiveness, respecting personal preferences, and combining non-pharmacological treatments with pharmacotherapy for a more comprehensive approach [80].

#### 8.2.1. Diet and Lifestyle Modifications

Lifestyle modifications play a significant role in migraine prevention and management, focusing on the changes that reduce attack frequency and severity without side effects [123]. Key strategies include identifying and avoiding migraine triggers, with alcohol being a significant one, and making broader lifestyle changes like increasing physical activity, managing weight, and improving sleep [123,124]. Regular exercise is recommended to reduce migraine frequency and severity [123,124]. Diet represents a significant factor in preventing and treating headaches, especially migraines. Certain foods, like caffeine, alcohol, aged cheeses, cured meats, and foods with artificial sweeteners or preservatives, can trigger headaches. Restricting these foods, especially in migraine-prone individuals, may help reduce headache frequency. Obesity is also linked to migraines, and weight loss can improve symptoms. Furthermore, a ketogenic diet (high-fat, low-carb) has shown some promise in reducing migraine frequency and severity, though more research is needed to confirm its effectiveness and identify who might benefit most [80,123,124]. Magnesium supplementation has also been shown to play a role in preventing migraines, with several studies suggesting that adequate magnesium levels may help reduce the frequency and intensity of attacks, particularly in individuals with low magnesium levels [125]. Riboflavin (vitamin B2) has similarly been identified as a beneficial supplement, with research indicating that it can reduce the frequency and duration of migraines, likely by improving mitochondrial energy metabolism [126].

#### 8.2.2. Biofeedback and Relaxation Techniques

Biofeedback has proven to be an effective method for managing migraines, as it positively influences autonomic nervous system activity and enhances resilience to the stressors that could precipitate migraine episodes [127]. Biofeedback uses specialized equipment to translate the physiological signals into visual or auditory cues, which are displayed on a screen. Guided by a trained practitioner, patients learn to regulate their physiological responses, much like using a mirror to adjust movements or expressions [128]. Various forms of biofeedback, including electromyographic (EMG), thermal, and heart rate variability (HRV) biofeedback, focus on different physiological processes such as muscle tension, blood circulation, and the regulation of the autonomic nervous system [129]. This method is often effective in treating chronic headaches, particularly migraines and TTH [130], as a study found that 70% of participants experienced a reduction of at least 50% in the frequency of their headaches, with the improvement lasting an average of 14.5 months after the therapy ended [131]. However, the use of biofeedback remains somewhat limited, primarily due to the need for costly equipment and trained professionals [127].

Yoga and meditation have also been studied for their potential therapeutic benefits in managing chronic conditions, including stress, anxiety, and migraines. A recent study found that an integrated education and relaxation program incorporating yoga and meditation significantly reduced the frequency, severity, and duration of migraine attacks, as well as improved quality of life [132]. Another study examined the impact of mindfulness-based stress reduction (MBSR) on migraine frequency and pain perception, with the results showing no significant reduction in migraine days but improved pain intensity and unpleasantness. This suggests that MBSR may offer benefits in pain management [133]. Both practices foster a mind–body connection that can contribute to overall well-being and may help reduce reliance on pharmacological interventions in migraine management. The combination of a therapeutic educational approach and progressive muscle relaxation, alongside standard pharmacological therapy, significantly improves the clinical outcomes in patients with migraines by reducing the intensity, frequency, and duration of migraines, as well as enhancing the quality of life [134].

#### 8.2.3. Cognitive-Behavioral Therapy (CBT) and Acupuncture

Cognitive-behavioral therapy (CBT) is based on the premise that negative thought patterns sustain mental disorders and psychological distress [135]. It aims to alter these maladaptive thoughts to improve emotional well-being and behavior [135]. CBT is particularly effective for managing migraines and tension-type headaches, which are often linked to emotional and psychological factors [135]. A recent study found that 83% of pediatric patients who completed a short course of CBT experienced a reduction in migraine frequency [136]. Additionally, newer research demonstrated that ten sessions of transdiagnostic CBT (TCBT) significantly improved migraine severity, disability, and associated anxiety and depression, with the effects lasting for one month after treatment [137]. CBT can also reduce headache frequency in patients with migraines, contributing to overall migraine management [138].

Acupuncture has been increasingly explored as a treatment for migraines, with multiple studies suggesting its effectiveness in reducing both the frequency and intensity of migraine attacks [139]. A systematic review found that acupuncture could reduce headache frequency, with up to 59% of participants experiencing a 50% or greater reduction in migraine occurrences. These benefits may persist for more than six months after treatment [140]. A German randomized controlled trial showed that 11 acupuncture sessions over six weeks were as effective as daily β-blocker treatment over six months [141]. This study, along with others, contributed to a 2009 Cochrane review that concluded acupuncture is beneficial for treating acute migraine attacks and is at least as effective as prophylactic drug treatments, with fewer adverse effects [142]. Moreover, research suggests that true acupuncture may lead to a long-term reduction in migraine recurrence compared to sham acupuncture or a waiting list [143].

#### 8.2.4. Physical Therapy

Physical therapy and relaxation techniques have been shown to be effective in managing both tension-type headaches (TTH) and migraines by targeting the underlying physical and psychological factors [144]. Although there is no standardized protocol for physical therapy in TTH, techniques aimed at the cranio-cervical-mandibular region, such as manual therapy, myofascial release, and relaxation techniques, have been shown to significantly reduce pain intensity and headache frequency [145]. Physical therapy often includes cervical manipulation and mobilization, soft tissue treatments (such as compression and massage), therapeutic exercises, and needling techniques to address musculoskeletal issues that may contribute to headache symptoms [80].

#### 8.2.5. Neurostimulation

Neuromodulation techniques, such as transcranial magnetic stimulation (TMS), transcranial direct current stimulation (tDCS), and peripheral nerve stimulation (PNS), have been explored as non-pharmacological treatments for migraines. Studies suggest that TMS may reduce migraine severity and frequency, though the evidence is limited due to methodological flaws and heterogeneity [146]. Similarly, tDCS has shown the potential to decrease the number of migraine days per month and pain intensity, with a good safety profile [147]. Peripheral nerve stimulation, particularly of the occipital nerves, has demonstrated efficacy in reducing headache pain and the disability associated with chronic migraines over a 12-month period [148]. While these findings are promising, further research with standardized protocols and larger sample sizes is needed to fully establish the effectiveness and safety of these neuromodulation therapies for migraine management.

Vagus nerve stimulation (VNS) has emerged as a promising therapeutic approach for stress-induced headaches, including TTH and migraines [149]. Invasive VNS modalities involve implanting a device that delivers electrical impulses to the vagus nerve, which has been associated with significant reductions in migraine attack frequency and severity [149,150]. Non-invasive VNS (nVNS) offers a less invasive alternative, using external devices to stimulate the vagus nerve. The gammaCore device, a handheld nVNS device, is FDA-cleared for the acute and preventive treatment of primary headaches, including episodic and chronic migraines [151]. Clinical trials have demonstrated that nVNS significantly reduces both pain intensity and frequency in patients with these conditions. Furthermore, nVNS has been shown to decrease headache days, reduce acute medication use, and improve headache-related disability and quality of life, supporting its role as an effective therapeutic option for primary headaches [152,153,154].

Overall, both invasive and non-invasive VNS have shown promise in the treatment of stress-induced headaches. While non-invasive VNS devices, such as gammaCore, provide a less invasive option with proven efficacy, invasive VNS may be considered for patients with refractory headaches unresponsive to other treatments. Further large-scale, controlled studies are needed to fully elucidate the mechanisms and optimize the use of VNS in headache management [150,151,152,153,154,155].

## 9. Conclusions

Chronic stress plays a critical role in the onset and progression of headache disorders, primarily through the dysregulation of the hypothalamic–pituitary–adrenal (HPA) axis and the autonomic nervous system (ANS). This dysregulation contributes to heightened pain sensitivity, neuroinflammation, and vascular changes, which exacerbate headache frequency and severity. The interplay between chronic stress and headaches establishes a bidirectional cycle, where stress triggers headaches, and recurrent headaches further amplify the stress responses.

Pharmacological treatments remain a cornerstone of headache management; however, addressing the underlying stress-related mechanisms may provide a more sustainable approach. Non-pharmacological strategies such as cognitive-behavioral therapy, biofeedback, relaxation techniques, and lifestyle modifications offer promising results in mitigating stress and reducing headache severity.

Further research is warranted to explore the long-term efficacy and optimization of these approaches. A deeper understanding of the physiological mechanisms underpinning the HPA axis and ANS dysregulation may pave the way for targeted interventions that not only alleviate symptoms but also address the root causes of stress-induced headache disorders. This comprehensive approach has the potential to improve both clinical outcomes and quality of life for individuals affected by chronic headaches.

## Figures and Tables

**Table 1:** Prevalence of primary headaches worldwide.

Type of Headache	Prevalence
Migraines	14%
Tension-Type Headaches	26%
Cluster Headaches	0.1–0.2%
Chronic Headaches	4.6%

**Table 2:** Risk factors and clinical features of primary headaches.

Type of Headache	Risk Factors	Clinical Features
Tension-Type Headache (TTH)	Psychosocial stress; Poor posture; Physical inactivity; Sleep disturbances; Anxiety; Depression; Excessive screen time [87].	Recurrent mild to moderate bilateral pain, often described as a pressing or tightening sensation, without nausea or heightened sensitivity to light/sound [108].
Migraine	Acute medication overuse; Female gender; Hormonal changes; Stress; Sleep deprivation; Certain foods; Environmental stimuli [109].	Intense throbbing pain, often unilateral, accompanied by nausea, vomiting, and photophobia/phonophobia, with or without aura [96].
Migraine with Coexisting Tension-Type Headaches (MigTTH)	Chronic stress; Sleep disturbances; Psychiatric comorbidities; Tension-type headache history [107].	Combination of pulsating, unilateral pain of migraines and bilateral, pressing pain of TTH [105].
Cluster Headache	Disruptions in circadian rhythms hypothalamic dysfunction; Smoking; Alcohol; Genetic predisposition; Seasonal changes; Vasodilators [83].	Excruciating unilateral pain around the eye or temple, often described as piercing or burning. Accompanied by autonomic symptoms (lacrimation, nasal congestion, eyelid swelling, facial sweating). Attacks occur in clusters over weeks or months, separated by remission periods [83].
